# The causal relationship between sleep disturbances and the risk of frailty: a two-sample Mendelian randomization study

**DOI:** 10.1007/s10433-024-00804-2

**Published:** 2024-03-19

**Authors:** Zong-Xiao Lu, Ni Sang, Rong-Chao Liu, Bo-Han Li, Meng-Yao Zhang, Ming-Hui Zhang, Meng-Cheng Cheng, Guo-Cui Wu

**Affiliations:** School of Nursing, Anhui Medical University, 15 Feicui Road, Hefei, Anhui China

**Keywords:** Mendelian randomization study, Causal relationship, Frailty, Sleep disturbances

## Abstract

**Objective:**

Adequate sleep is closely related to people's health. However, with increasing age, the quality of sleep worsens. At the same time, among elderly individuals, frailty is also a disturbing factor, which makes elderly individuals more vulnerable to negative factors. To explore the relationship between the two, we conducted this study.

**Methods:**

In this paper, independent genetic variations related to insomnia, sleep duration and daytime sleepiness were selected as IVs, and related genetic tools were used to search published genome-wide association studies for a two-sample Mendelian randomization (TSMR) analysis. The inverse-variance weighted (IVW) method was used as the main Mendelian randomization analysis method. Cochran's Q test was used to test heterogeneity, MR‒Egger was used to test horizontal pleiotropy, and the MR-PRESSO test was used to remove outliers.

**Results:**

According to our research, insomnia (OR = 1.10, 95% CI 1.03–1.17, *P* = 2.59e−97), long sleep duration (OR = 0.66, 95% CI 0.37–1.17, *P* = 0.02), short sleep duration (OR = 1.30, 95% CI 1.22–1.38, *P* = 2.23e−17) and daytime sleepiness (OR = 1.49, 95% CI 1.25–1.77, *P* = 0.96e−4) had a bidirectional causal relationship with frailty.

**Conclusions:**

Our research showed that there is a causal relationship between sleep disturbances and frailty. This result was obtained by a TSMR analysis, which involves the use of genetic variation as an IV to determine causal relationships between exposure and outcome. Future TSMR studies should include a larger sample for analysis.

**Supplementary Information:**

The online version contains supplementary material available at 10.1007/s10433-024-00804-2.

## Introduction

Frailty is a clinical state in which individuals are more likely to experience negative health-related events (including falls, disability, hospitalization, fractures, and death) when exposed to stressors (Cesari et al. 2017; Cheung et al. 2020). This suggests that frail people will be more vulnerable than healthy people under the same stressors, and they may experience more serious consequences. Because the physiological reserves of a person continue to diminish during the ageing process, frailty is also common in elderly individuals, with an overall prevalence of 17% in elderly individuals in low- and middle-income countries. In addition, frailty can cause sleep problems in older adults, and studies have shown that patients with frailty are more likely than nonfrail individual to have sleep problems, such as difficulty falling asleep, interrupted sleep, and sleep duration ≤ 5 h (Lee et al. 2018).

Sleep is an active state in which the arousal threshold of a person in sleep is increased and the responsiveness to external stimuli is reduced (Besedovsky et al. 2019). Adequate sleep promotes recovery, eliminates fatigue, consolidates new skills, improves immunity and promotes physical development. Decreased sleep quality leads to emotional impairment, physical fatigue and cognitive impairment (Troynikov et al. 2018). Ageing is an irreversible process, and as age increases, the quality of human sleep decreases (Nobrega et al. 2014). Sleep disturbances are very common among older adults in the community, with 9–50% of older adults having sleep disturbances such as insomnia and a short sleep duration (Moreno-Tamayo et al. 2017; Vaz Fragoso et al. 2009). Sleep disturbances such as insomnia and daytime sleepiness often lead to symptoms such as fatigue, which in turn is a major feature of frailty (Pourmotabbed et al. 2020).

In a systematic review and meta-analysis, it was suggested that sleep duration < 6 h and daytime sleepiness both contributed to the development of frailty (Pourmotabbed et al. 2020). Poor sleep quality is a preventable and reversible risk factor for the development of frailty; the vicious cycle of poor sleep quality promotes the development of frailty in older adults, and frailty further contributes to the exacerbation of sleep disturbances (Wai and Yu 2020). At the same time, frailty leads to a decrease in social and exercise activities, and the loss of these age-old activities will lead to a high degree of irregularity in sleep, resulting in sleep disturbances, a phenomenon that is very common in elderly individuals (Vaz Fragoso et al. 2009). This evidence suggests that there is a relationship between sleep and frailty that can be explored, but there is no definitive evidence to prove the relationship. Therefore, we now use two-sample Mendelian randomization (TSMR) to explore whether sleep disturbances have a bidirectional causal relationship with frailty in terms of insomnia, sleep duration, and daytime sleepiness.

Mendelian randomization (MR) is a commonly used inferential method for assessing potential causal relationships between exposure factors and outcome variables using genome-wide summary association statistics, usually single-nucleotide polymorphisms (SNPs), as instrument variables (IVs) in epidemiological studies(Zhuang et al. 2021).

## Materials and methods

### Data sources

The data related to frailty were obtained from a large genome-wide association study (GWAS) that included 104,610 UK individuals (Atkins et al. 2021).

The common symptoms of insomnia include decreased sleep duration, decreased sleep quality and difficulty falling asleep. The data related to insomnia came from 57 self-reported insomnia loci in UK Biobank (n = 453379) (Lane et al. 2019).

The sleep duration data were obtained from a GWAS with a European population of 446118. A sleep duration < 6 h is defined as a short sleep duration, whereas a long sleep duration is defined as a sleep duration > 9 h and a normal sleep duration is 7–8 h (Dashti et al. 2019).

Daytime sleepiness varies considerably among individuals, and data related to daytime sleepiness were obtained from the GWAS containing 452,071 European populations (Wang et al. 2019).

As this study was based on published data, no ethical approval or informed consent was needed.

### Selection of instrumental variables

IVs in MR are subject to three assumptions: 1: IVs are to be correlated with exposure factors; 2: IVs do not affect outcome variables through pathways other than exposure; and 3: IVs are not correlated with confounders (Hemani et al. 2018). To make the IVs consistent with the assumptions of Mendelian randomization, we first extracted SNPs with *P* < 5e−8 from the data, after which we excluded linkage disequilibrium (LD) SNPs from our extracted SNPs (r^2^ < 0.001, clumping window = 10000 kb) to ensure that the exposed instruments were independent. We then proceeded to extract the SNPs associated with the outcome variables, and for missing SNPs, we searched for complementary proxy SNPs (r^2^ > 0.8). Finally, we harmonized the exposure data as well as the outcome data to exclude palindromic SNPs. Because the data were compared many times, we performed Bonferroni correction on the *P* value obtained from the final MR analysis, and the corrected *P* value was used to assess the statistical significance of the final result.

### TSMR analysis

We used inverse-variance weighted (IVW), MR‒Egger, weighted median, simple mode and weighted mode methods for TSMR, and the IVW method was used as the main MR method.

### Sensitivity analysis

For the assessment of heterogeneity, we used Cochran's Q test, the MR‒Egger and MR-PRESSO tests to detect pleiotropy and the MR-PRESSO test to remove outliers. The F statistic is calculated as F = R^2^ (n − k − 1)/[k (1 − R^2^)]. Generally, IVs are considered to be strong IVs as long as F statistics are greater than 10.

### Statistical analysis

We used R 4.2.0 for data analysis. The Two Sample MR package was used for TSMR, and the MR-PRESSO package was used for the MR-PRESSO test.

## Results

The source data used in this study are shown in Additional file 1: Tables S1–S6. The F statistics of insomnia, sleep duration, long sleep duration, short sleep duration and daytime sleepiness were 15.65, 11.89, 12.25, 14.23 and 18.62, respectively. When frailty was the exposure and insomnia was the outcome, the F statistic was 57.070; when sleep duration was the outcome, the F statistic was 53.778; and the remaining F statistic was 50.546. This means that there were no weak IVs. The variances explained by these IVs were sleep duration 0.09%, short sleep duration 0.06%, long sleep duration 0.02%, insomnia 0.12%, and daytime sleepiness 0.13%.

### Bidirectional causality of insomnia and frailty

In the study of the effect of insomnia on frailty risk, At first we extracted 2500 SNPs (p<5e-8), and 48 SNPs remained after performing LD. After harmonizing exposure data and outcome data, 45 SNPs remained. MR-PRESSO testing showed 10 SNPs with pleiotropy, and after removal 35 SNPs remained as the final IVs for MR analysis. The IVW model (OR = 2.32, 95% CI 2.14-2.51, *P* = 2.59e-97) showed a causal relationship between insomnia and frailty (Table 1). The MR‒Egger regression test (intercept = 0.13e−2, *P* = 0.64) did not show any evidence of directional pleiotropy. Cochran’s Q test showed strong heterogeneity among the IVs (*P* = 3.49e−5) (Additional file 1: Table S7).

**Table 1:** MR analysis for the causality of sleep disturbances with the risk of frailty

Exposure/outcome	Nsnp	Methods	OR(95%CI)	SE	*P* value	*P(Bonferroni correction)*	Horizontal pleiotropy					Heterogeneity		
							MR-Egger regression			MR-PRESSO		Cochran’s Q	*P* value	F
							Egger intercept	SE	*P* value	Global test *P* value	Distortion test *P*val			
Insomnia/frailty	35	IVW	2.32 (2.14–2.51)	0.04	2.59e−96	2.59 e−97	1.27 e−03	2.73 e−03	6.44 e−01	0.17	NA	77.04	3.49 e−05	15.65
		MR Egger	2.04 (1.18–3.52)	0.279	1.54 e−02	1.54 e−03								
		Weighted median	2.16 (1.86–2.52)	0.08	1.17 e−23	1.17 e−25								
		Weighted mode	2.40 (1.82–3.16)	0.14	4.96 e−07	4.96 e−09								
Sleep duration/frailty	34	IVW	0.998 (0.998–0.999)	0.35 e−3	0.18 e−3	0.18 e−5	− 0.01	0.26 e−2	0.01	0.01	NA	67.26	0.26 e−3	11.89
		MR Egger	1.00 (1.00–1.01)	0.32 e−3	0.34 e−2	0.34 e−4								
		Weighted median	0.999 (0.998–0.999)	0.32 e−3	0.34 e−2	0.34 e−3								
		Weighted mode	0.999 (0.998–0.999)	0.27 e−3	0.33 e−2	0.33 e−3								
Long sleep duration/frailty	10	IVW	0.66 (0.37–1.17)	0.29	0.16	0.02	− 0.71 e−2	0.53 e−02	0.22	0.72	NA	6.29	0.71	12.25
		MR Egger	2.05 (0.35–11.93)	0.90	0.45	0.05								
		Weightedmedian	0.65 (0.32–1.33)	0.37	0.24	0.02								
		Weighted mode	0.62 (0.27–1.43)	0.43	0.29	0.03								
Short sleep duration/frailty	22	IVW	1.30 (1.22–1.38)	0.03	2.23 e−16	2.23 e−17	0.71 e−2	0.30 e−2	0.03	0.38	NA	234.91	4.33e− 38	14.23
		MR Egger	0.64 (0.35–1.14)	0.30	1.47 e−01	1.47 e−2								
		Weighted median	1.32 (1.24–1.40)	0.03	1.44 e−17	1.44 e−18								
		Weighted mode	1.29 (1.22–1.37)	0.26	1.78 e−08	1.78 e−09								
Daytime sleepiness/frailty	20	IVW	1.49 (1.25–1.77)	0.09	9.46 e−06	9.46 e−07	− 5.10 e−04	1.93 e−03	0.79	0.26	NA	24.15	0.19	18.62
		MR Egger	1.58 (0.96–2.61)	0.26	8.82 e−02	8.82 e−3								
		Weighted median	1.40 (1.12–1.75)	0.11	2.96 e−03	2.96 e−04								
		Weighted mode	1.34 (1.08–1.67)	0.11	1.54 e−02	1.54 e−3								

*MR-PRESSO* MR-Pleiotropy Residual Sum and Outlier method, *OR* odds ratio, *CI* confidence interval, *IVW* inverse-variance weighted

P.value is the *P* value before Bonferroni correction

We extracted 5252 SNPs (*P* < 5e−8) for the study of frailty on insomnia and 20 SNPs remained after performing LD. After harmonizing exposure data and outcome data,11 SNPs were used as IVs for MR (Additional file 1: Table S6). MR analysis IVW results (OR = 1.10, 95% CI 1.03–1.17 *P* = 0.28e−3) showed a causal relationship between insomnia and frailty. The MR‒Egger regression test (intercept = 0.01, *P* = 0.06) did not show any evidence of directional pleiotropy. The heterogeneity test showed the presence of heterogeneity in IVs (*P* = 0.01).

### Bidirectional causality of sleep duration and frailty

We extracted 7249 SNPs (*P* < 5e−8) for the study of sleep duration on frailty risk and 70 SNPs remained after performing LD , After harmonizing exposure data and outcome data , There were 66 SNPs remaining. MR-PRESSO testing showed 32 SNPs with pleiotropy, 34 IVs were ultimately used for MR analysis. The IVW model showed a causal relationship between sleep duration and frailty (OR = 0.998, 95% CI 0.998-0.999, *P* =0.18e-5). The MR‒Egger test showed evidence for directional pleiotropy (intercept = −0.006, *P* = 0.01), and Cochran's Q test showed heterogeneity for sleep duration (*P* = 0.002) (Additional file 1: Table S7).

We extracted 3900 SNPs (*P* < 5e−8) for the study of long sleep duration on frailty risk and 10 SNPs remained after performing LD. After harmonizing exposure data and outcome data 10 SNPs remained. The IVW model showed no causal relationship between long sleep duration (OR = 0.66, 95% CI 0.37–1.17, *P* = 0.02) and frailty. The MR-PRESSO test (*P* = 0.52) showed no pleiotropy. The MR‒Egger regression test (intercept = -0.01, *P* = 0.17) did not show any evidence of directional pleiotropy, and Cochran's Q test showed (*P* = 0.51) no heterogeneity (Additional file 1: Table S7).

We extracted 861 SNPs (*P* < 5e−8) for the study of short sleep duration on frailty risk and 26 SNPs remained after performing LD. After harmonizing exposure data and outcome data, the remaining 21 SNPs were included in the final MR analysis. IVW (OR = 1.30, 95% CI 1.22–1.38, *P* =2.23e−17) indicated a causal relationship between short sleep duration and frailty (Table 1). The MR-PRESSO test showed no pleiotropy (*P* = 0.38), the MR‒Egger regression test showed directional pleiotropy (intercept = −0.01, *P* = 0.03), and Cochran's Q test showed heterogeneity (*P* = 4.33e−38) (Additional file 1: Table S7),

We extracted 5252 SNPs (*P* < 5e−8) for the study of frailty on sleep duration risk and 20 SNPs remained after performing LD, After harmonizing exposure data and outcome data 13 SNPs remained. The IVW model showed no causal relationship between frailty and sleep duration (OR = 0.99, 95% CI 0.94–1.06,* P* = 0.10). The MR-PRESSO test showed the presence of pleiotropy in rs8089807. After its removal, the MR-PRESSO test showed no pleiotropy (*P* = 0.46), and the MR‒Egger regression test did not show any evidence of directional pleiotropy (intercept = −0.004, *P* = 0.21). Cochran’s Q test showed no heterogeneity among the IVs (*P* = 0.44).

After LD and harmonizing, there were 13 SNPs as IVs that have been used in studies of long sleep time and frailty. The IVW model showed no causal relationship between frailty and long sleep duration (OR = 1.03, 95% CI 1.01–1.06,* P* = 0.98e−3). The MR-PRESSO test showed no pleiotropy (*P* = 0.15). Cochran’s Q test (P=0.09) and the pleiotropy test (intercept = 6.95e−3, *P* = 0.55) showed no heterogeneity or pleiotropy.

In our study, After LD and harmonizing, 13 SNPs that were strongly associated with frailty were included in the final MR analysis. The IVW model showed no causal relationship between frailty and short sleep duration (OR = 1.05, 95% CI 1.01–1.08, *P* = 0.52e−3). The MR-PRESSO test showed the absence of pleiotropy (*P* = 0.17). The MR‒Egger regression test did not show any evidence of directional pleiotropy (intercept = 0.001, *P* = 0.33). Cochran’s Q test showed no heterogeneity among the IVs (*P* = 0.10) (Additional file 1 Table S7).

### Bidirectional causality of daytime sleepiness and frailty

We extracted 5657 SNPs (*P* < 5e−8) for the study of daytime sleepiness on frailty risk and 38 SNPs remained after performing LD, After harmonizing exposure data and outcome data 37 SNPs remained MR-PRESSO testing showed 17 SNPs with pleiotropy, 20 SNPs that were strongly associated with daytime sleepiness were included in the final MR analysis. The IVW model showed a causal relationship between daytime sleepiness and frailty (OR = 1.49, 95% CI 1.25–1.77, *P* = 9.46e−7) (Table 1). Cochran’s Q test showed no heterogeneity (*P* = 0.19), and the pleiotropy test showed no pleiotropy (*P* = 0.80) (Additional file 1 Table S7).

In the study of the effect of frailty on daytime sleepiness risk, After LD and harmonizing, we extracted 13 SNPs. The IVW model showed a causal relationship between the two (OR = 1.06, 95% CI 1.02−1.10, *P* = 0.37e−3) (Table 1). The MR-PRESSO test showed no pleiotropy among the IVs (*P* = 0.11), while Cochran's Q test showed no heterogeneity among the SNPs (*P* = 0.05), and the pleiotropy test showed no pleiotropy among the IVs (intercept = 0.001, *P* = 0.44) (Additional file 1 Table S7).

## Discussion

We conducted a TSMR study to investigate whether each sleep characteristic had a two-way causal relationship with frailty. According to our study, a long sleep duration is causally related to frailty, but frailty does not cause a long sleep duration. Insomnia, short sleep duration and daytime sleepiness have a bidirectional causal relationship with frailty (Table 2).

**Table 2:** MR analysis of the causal relationship between frailty and the risk of sleep disturbances

Exposure/outcome	Nsnp	Methods	OR (95%CI)	SE	*P* value	*P(Bonferroni correction)*	Horizontal pleiotropy					Heterogeneity		
							MR-Egger regression			MR-PRESSO		Cochran’s Q	*P* value	F
							Egger intercept	SE	*P* value	Global test *P* value	Distortion test *P*val			
Frailty/Insomnia	11	IVW	1.10 (1.03–1.17)	0.03	0.28e−2	0.28e−3	4.93e−03	2.29e−03	0.06	0.07	NA	22.98	0.01	57.07
		MR Egger	0.90 (0.74–1.09)	0.10	0.29	0.03								
		Weighted median	1.06 (0.99–1.13)	0.03	0.07	0.71e−2								
		Weighted mode	1.04 (0.97–1.12)	0.04	0.33	0.03								
Frailty/sleep duration	12	IVW	0.99 (0.94–1.06)	0.03	0.95	0.10	− 3.65e−03	2.73e−03	0.21	0.46	NA	11.03	0.44	53.78
		MR Egger	1.17 (0.92–1.48)	0.12	0.233	0.02								
		Weighted median	1.01 (0.92–1.11)	0.05	0.83	0.08								
		Weighted mode	1.01 (0.89–1.14)	0.07	0.93	0.09								
Frailty/Long sleep duration	13	IVW	1.03 (1.01–1.06)	0.01	0.98e−2	0.98e−3	6.95e−03	0.11e−2	0.55	0.15	NA	18.97	0.09	50.55
		MR Egger	1.00 (0.91–1.11)	0.05	0.95	0.01								
		Weighted median	1.03 (1.00–1.06)	0.02	0.06	0.01								
		Weighted mode	1.03 (0.99–1.08)	0.02	0.20	0.02								
Frailty/Short sleep duration	13	IVW	1.05 (1.013–1.080)	0.02	0.01	0.52e−3	1.470e−03	1.440e−03	0.33	0.17	NA	18.56	0.10	50.55
		MR Egger	0.98 (0.87–1.11)	0.06	0.80	0.08								
		Weighted median	1.03 (0.99–1.07)	0.02	0.16	0.02								
		Weighted mode	1.03 (0.97–1.09)	0.03	0.38	0.04								
Frailty/Day time sleepiness	13	IVW	1.06 (1.02–1.10)	0.02	0.37e−2	0.37e−3	1.35e−03	1.67e−03	0.44	0.11	NA	21.05	0.05	50.55
		MR Egger	0.99 (0.86–1.15)	0.07	0.97	0.10								
		Weighted median	1.05 (1.01–1.09)	0.02	0.02	0.15e−2								
		Weighted mode	1.05 (0.99–1.11)	0.03	0.14	0.01								

*MR-PRESSO* MR-Pleiotropy Residual Sum and Outlier method. *OR* odds ratio; *CI* confidence interval; *IVW* inverse-variance weighted

P.value is the *P* value before Bonferroni correction

### Insomnia and frailty

Our study found that insomnia is a risk factor for frailty. Previous studies have shown a causal relationship between insomnia and frailty after adjusting statistical models for sociodemographic and health covariates (Ensrud et al. 2012; Moreno-Tamayo et al. 2020; Nemoto et al. 2021). In a US survey, chronic body pain, depression and other problems were found to be associated with insomnia (Foley et al. 2004). Insomnia was usually accompanied by depression, and at the same time, depression was a correlate of frailty, which further suggests that insomnia results in frailty (Dragioti et al. 2018; Lee et al. 2018; Liu et al. 2021).

### Sleep duration and frailty

Our research has revealed a bidirectional causal relationship between short sleep duration and frailty. An association between short sleep duration and frailty after controlling for various covariates was also reported in a previous study, which is consistent with our analysis; people with long sleep duration have lower activity levels and more fatigue symptoms than the rest of the population (Baniak et al. 2020; Moreno-Tamayo et al. 2021; Nakakubo et al. 2018). Since a low level of physical activity was shown to be a cause of frailty, we believed that the association between long sleep duration and frailty was caused by low levels of physical activity. In addition, because people with frailty are more easily fatigued, they may need to sleep for long periods of time to reduce their fatigue, so long sleep duration may be a compensatory activity for people with frailty to reduce fatigue (Jike et al. 2018).

### Daytime sleepiness and frailty

Our findings showed a bidirectional causal relationship between daytime sleepiness and frailty. A possible bidirectional relationship between daytime sleepiness and debilitation was suggested in previous studies, and after correcting for various covariates, a relationship between daytime sleepiness and the onset of debilitation was found(Ensrud et al. 2009; Vaz Fragoso et al. 2009).

In conclusion, our study demonstrates that sleep disturbances such as insomnia, short sleep duration, and daytime sleepiness are causally related to frailty. However, the mechanisms between them are poorly understood, and some possible pathways involve the role of adipokines. In previous studies on adiponectin and sleep disturbances, it was found that plasma adiponectin levels remained strongly correlated with sleep disturbances after adjusting for various confounding factors and that elevated adiponectin was closely related to sleep disturbances (Pourmotabbed et al. 2020; Zeng et al. 2017). Adiponectin plays a significant role in weight loss and muscle loss and is significantly negatively correlated with muscle mass and muscle density; therefore, higher levels of adiponectin in the body are associated with less muscle, which is a feature of frailty (Arai et al. 2019; Ma et al. 2018; Nagasawa et al. 2018). Leptin is an adipokine. It has been shown that serum leptin is increased in patients with frailty, and the increase in leptin can infiltrate skeletal muscle, leading to muscle damage and thus increasing the risk of frailty (Lana et al. 2017). Likewise, leptin acts on the hypothalamus, thus keeping sleep normal in humans. Patients with increased leptin levels can suffer from severe sleep deprivation (Mosavat et al. 2021). Therefore, adipokines play an important role in frailty and sleep disturbances.

Glucocorticoids affect the activity of skeletal muscle, bone and other metabolic tissues, and the development of frailty is closely related to these tissues (Clegg and Hassan-Smith 2018). Low secretion of cortisol in glucocorticoids causes disorders of the hypothalamic-pituitary-adrenocortical hormone axis and sleep-adrenal insufficiency, resulting in severe fatigue, drowsiness and poor sleep quality (Morgan and Tsai 2015). Excess thyroid hormone also tends to cause patients to be more irritable and increase the incidence of restless legs syndrome, which in turn can exacerbate difficulties with falling asleep and insomnia (Morgan and Tsai 2015). Thus, thyroid hormones may be one way in which frailty has a causal relationship with sleep disturbances. In a cross-sectional study with 3943 participants, elevated serum FT4 concentrations were also found to be associated with an increased risk of frailty in men (Clegg and Hassan-Smith 2018).

IL-6 is a proinflammatory cytokine in human tissues that induces apoptosis in muscle tissue, leading to myocyte death. TNF-α, on the other hand, can inhibit skeletal muscle contraction through endocrine effects. IL-6, CRP and TNF-α are significantly increased in different regions of the body in frailty and prefrailty patients and are independent risk factors for frailty(Clegg et al. 2013; Picca et al. 2022; Soysal et al. 2016; Xu et al. 2022). In addition, inflammatory cytokines can regulate sleep rhythm (Besedovsky et al. 2019). When sleep deprivation occurs, IL-6 and TNF-α increase in the body, and when the body shows high levels of inflammation, it leads to a decrease in sleep duration and induces split sleep, which leads to sleep disturbances such as insomnia (Irwin 2019).

Our study has several advantages. (1) To our knowledge, it was the first to use Mendelian randomization to analyse the relationship between frailty and sleep. MR can largely avoid confounding and reverse causality and is easier to perform. (2) We extracted our data from a GWAS with a large number of clinical samples, thus ensuring that our sample size was sufficient to effectively avoid bias caused by a small sample size.

In addition, this study has some limitations. First, because aggregated data were used, stratification effects could not be performed to explore the differences between sexes. Second, our sample data were collected mainly from the European population, so the study is less suitable for generalization to other regions and requires caution in its application. In addition, direct translation of genetic effect sizes to clinical intervention effects is not feasible because genetic effects occur in the body from the beginning of life, and therefore, the results obtained from TSMR analyses usually have larger effect sizes than the results of clinical interventions. Finally, the sleep characteristics in this study were self-reported and therefore may be subject to some measurement error.

In summary, our research showed that there is a two-way relationship between insomnia, daytime sleepiness and frailty. Future TSMR studies should include a larger sample for analysis.

### Supplementary Information

**Additional file 1. **Supplementary tables and figures.
