# Supplementary material for: The causal relationship between sleep disturbances and the risk of frailty: a two-sample Mendelian randomization study
Source: Eur J Ageing. 2024 Mar 19;21(1):9. doi: 10.1007/s10433-024-00804-2 (PMC10951186; doi:10.1007/s10433-024-00804-2)
Supplement: Supplementary file 1 — Additional file 1. Supplementary tables and figures. [file 10433_2024_804_MOESM1_ESM.docx]

**Supplementary materials**

**Supplementary Table 1.** The summary information for instrumental variables of insomnia.

| SNP | effect_allele | other_allele | beta | eaf | se | pval |
| --- | --- | --- | --- | --- | --- | --- |
| rs10156602 | A | G | 0.0101122 | 0.637771 | 0.00144819 | 3.40E-12 |
| rs1031654 | C | A | 0.0102622 | 0.201391 | 0.00172522 | 2.00E-09 |
| rs11097861 | A | G | -0.00916107 | 0.284352 | 0.00153452 | 1.70E-09 |
| rs11184946 | C | T | -0.00878653 | 0.582984 | 0.00139968 | 2.90E-10 |
| rs11635495 | T | C | -0.00813358 | 0.485702 | 0.00138626 | 6.80E-09 |
| rs11673344 | A | G | -0.00868506 | 0.619812 | 0.00142888 | 9.00E-10 |
| rs11804386 | G | A | -0.00805764 | 0.666568 | 0.00146423 | 2.60E-08 |
| rs12405761 | A | C | 0.00923926 | 0.570539 | 0.00139853 | 2.60E-11 |
| rs1430205 | C | T | -0.00769252 | 0.541669 | 0.0013907 | 3.60E-08 |
| rs1544637 | T | C | 0.00767466 | 0.487251 | 0.00138704 | 3.00E-08 |
| rs17139246 | T | C | -0.00777532 | 0.610884 | 0.00142927 | 4.10E-08 |
| rs17151854 | G | T | -0.010705 | 0.846784 | 0.00192286 | 2.40E-08 |
| rs17669584 | A | G | -0.00964647 | 0.80454 | 0.00178391 | 3.60E-08 |
| rs1942262 | G | A | -0.0111508 | 0.707539 | 0.00152288 | 1.10E-13 |
| rs2062113 | T | C | 0.00889979 | 0.429456 | 0.00140332 | 1.90E-10 |
| rs2296580 | G | T | 0.0103125 | 0.702012 | 0.00151028 | 8.70E-12 |
| rs2297787 | T | A | 0.0152861 | 0.920608 | 0.00256133 | 2.10E-09 |
| rs28061 | A | G | 0.00836258 | 0.692321 | 0.00150548 | 2.10E-08 |
| rs2956278 | A | G | -0.00952347 | 0.785194 | 0.00168476 | 1.30E-08 |
| rs3104778 | A | G | 0.0078046 | 0.589631 | 0.00141099 | 4.20E-08 |
| rs314280 | A | G | -0.00863865 | 0.453284 | 0.00138926 | 3.50E-10 |
| rs35881094 | T | G | -0.0110528 | 0.573415 | 0.00140168 | 3.00E-15 |
| rs3824081 | T | C | 0.00801355 | 0.475638 | 0.0013892 | 1.10E-08 |
| rs4577309 | A | G | 0.00811816 | 0.46879 | 0.00138836 | 3.70E-09 |
| rs4688760 | C | T | -0.0107674 | 0.309505 | 0.00149946 | 1.00E-12 |
| rs4751 | G | T | -0.00772701 | 0.575309 | 0.00139656 | 1.60E-08 |
| rs4886860 | G | C | 0.0112372 | 0.234173 | 0.00163281 | 6.10E-12 |
| rs6593005 | A | G | -0.00903102 | 0.258967 | 0.00157978 | 8.60E-09 |
| rs6664467 | G | A | 0.0112654 | 0.86353 | 0.00201555 | 4.50E-08 |
| rs68094047 | C | T | -0.00941756 | 0.749565 | 0.00160041 | 3.30E-09 |
| rs6932158 | T | C | -0.00776019 | 0.509426 | 0.00138432 | 2.80E-08 |
| rs7711696 | G | T | -0.0101773 | 0.695476 | 0.00150096 | 9.90E-12 |
| rs79780963 | C | T | 0.0148998 | 0.922913 | 0.00257912 | 6.30E-09 |
| rs9845387 | C | A | 0.0196529 | 0.959258 | 0.00350129 | 2.10E-08 |
| rs9894577 | G | A | -0.0123638 | 0.682406 | 0.00148745 | 1.50E-16 |

**Supplementary Table 2.** The summary information for instrumental variables of sleep duration

| SNP | effect_allele.exposure | other_allele.exposure | beta.exposure | eaf.exposure | se.exposure | pval.exposure |
| --- | --- | --- | --- | --- | --- | --- |
| rs10173260 | C | T | 0.665 | 0.606 | 0.1514898 | 1.20E-05 |
| rs10761674 | C | T | 0.739 | 0.477 | 0.1481418 | 5.10E-07 |
| rs10973207 | T | G | 0.997 | 0.158 | 0.2043294 | 1.20E-06 |
| rs113113059 | T | C | 0.984 | 0.78 | 0.1793076 | 6.60E-08 |
| rs11602180 | C | T | 1.045 | 0.837 | 0.200682 | 1.90E-07 |
| rs11621908 | C | T | 1.511 | 0.917 | 0.272208 | 2.30E-08 |
| rs11643715 | G | C | 0.804 | 0.291 | 0.1632456 | 9.20E-07 |
| rs11885663 | T | C | 1.102 | 0.248 | 0.1712838 | 1.10E-10 |
| rs12607679 | T | C | 0.981 | 0.738 | 0.1697574 | 7.60E-09 |
| rs12791153 | T | A | 1.486 | 0.081 | 0.2758776 | 5.40E-08 |
| rs13109404 | T | G | 2.38 | 0.928 | 0.289245 | 1.90E-16 |
| rs1517572 | C | A | 0.669 | 0.581 | 0.150156 | 7.10E-06 |
| rs1939455 | G | T | 1.259 | 0.879 | 0.2329764 | 7.60E-06 |
| rs1991556 | G | A | 0.919 | 0.774 | 0.1781934 | 2.60E-07 |
| rs2072727 | T | C | 0.692 | 0.436 | 0.1495914 | 3.20E-06 |
| rs2079070 | C | G | 0.912 | 0.265 | 0.1676172 | 5.00E-08 |
| rs2192528 | A | G | 0.716 | 0.48 | 0.148398 | 1.00E-06 |
| rs2231265 | G | A | 0.861 | 0.772 | 0.1765674 | 1.00E-06 |
| rs3095508 | C | A | 0.914 | 0.594 | 0.1505694 | 1.40E-09 |
| rs330088 | C | T | 0.972 | 0.547 | 0.1489074 | 6.60E-11 |
| rs34556183 | A | G | 0.791 | 0.72 | 0.1648938 | 1.50E-06 |
| rs34731055 | T | C | 0.952 | 0.181 | 0.1923486 | 7.30E-07 |
| rs4128364 | C | T | 0.847 | 0.339 | 0.156411 | 6.60E-08 |
| rs4767550 | G | A | 0.873 | 0.414 | 0.151032 | 8.60E-09 |
| rs56372231 | T | C | 1.026 | 0.334 | 0.156903 | 6.30E-11 |
| rs61796569 | T | C | 0.899 | 0.27 | 0.1676082 | 3.20E-05 |
| rs6575005 | T | C | 0.957 | 0.758 | 0.1726914 | 4.10E-08 |
| rs72804080 | G | A | 1.036 | 0.15 | 0.209232 | 9.10E-07 |
| rs7644809 | T | C | 0.873 | 0.422 | 0.1506012 | 6.00E-09 |
| rs7806045 | T | C | 0.825 | 0.755 | 0.1719408 | 1.80E-06 |
| rs915416 | C | G | 1.096 | 0.29 | 0.163305 | 1.90E-11 |
| rs9345234 | C | A | 0.735 | 0.578 | 0.1503552 | 9.10E-07 |
| rs9382445 | T | C | 0.941 | 0.623 | 0.1527084 | 5.50E-10 |
| rs9940646 | C | G | 1.153 | 0.578 | 0.1498536 | 1.30E-14 |

**Supplementary Table 3.** The summary information for instrumental variables of long sleep duration

| SNP | effect_allele.exposure | other_allele.exposure | beta.exposure | eaf.exposure | se.exposure | pval.exposure |
| --- | --- | --- | --- | --- | --- | --- |
| rs10899257 | G | A | -0.00563809 | 0.855527 | 0.00103128 | 4.60E-08 |
| rs146467757 | G | T | 0.00563786 | 0.772153 | 0.000869897 | 8.10E-11 |
| rs147114641 | C | A | 0.00568908 | 0.77403 | 0.000867588 | 4.90E-11 |
| rs17817288 | A | G | 0.00418824 | 0.518127 | 0.000726521 | 8.90E-09 |
| rs3751046 | A | G | -0.00577495 | 0.852658 | 0.00102722 | 2.00E-08 |
| rs572857764 | T | A | 0.00562714 | 0.775271 | 0.000877581 | 1.30E-10 |
| rs62073915 | A | G | 0.00605568 | 0.77868 | 0.000891371 | 1.00E-11 |
| rs6737318 | A | G | -0.00638002 | 0.778159 | 0.000876506 | 3.40E-13 |
| rs7534398 | T | A | -0.00507915 | 0.798618 | 0.000908499 | 2.10E-08 |
| rs75458655 | C | T | -0.0167308 | 0.977027 | 0.00242295 | 5.40E-12 |

**Supplementary Table 4.** The summary information for instrumental variables of short sleep duration

| SNP | effect_allele.exposure | other_allele.exposure | beta.exposure | eaf.exposure | se.exposure | pval.exposure |
| --- | --- | --- | --- | --- | --- | --- |
| rs11763750 | G | A | 0.00721224 | 0.814346 | 0.00123366 | 5.10E-09 |
| rs1229762 | C | T | -0.00723933 | 0.335499 | 0.00101656 | 1.00E-12 |
| rs12518468 | T | C | -0.00588504 | 0.671544 | 0.00102098 | 8.50E-09 |
| rs12661667 | C | T | -0.00602193 | 0.736505 | 0.00108717 | 2.80E-08 |
| rs12963463 | C | T | 0.00711437 | 0.299425 | 0.0010601 | 1.90E-11 |
| rs13107325 | C | T | -0.0132682 | 0.925472 | 0.0018282 | 2.50E-13 |
| rs1380703 | A | G | -0.00676427 | 0.616469 | 0.0010046 | 1.60E-11 |
| rs1607227 | G | T | 0.0063693 | 0.704938 | 0.00105472 | 1.50E-09 |
| rs17005118 | G | A | -0.00648249 | 0.735064 | 0.00108701 | 2.50E-09 |
| rs17388803 | A | C | -0.00982621 | 0.894352 | 0.00158706 | 6.50E-10 |
| rs2014830 | C | T | 0.00578646 | 0.698128 | 0.00105038 | 2.70E-08 |
| rs2517827 | C | A | -0.00592859 | 0.688933 | 0.00103615 | 5.70E-09 |
| rs2820313 | A | G | -0.00600641 | 0.658888 | 0.00101025 | 2.30E-09 |
| rs2863957 | C | A | 0.0101896 | 0.781508 | 0.0011609 | 2.60E-18 |
| rs3776864 | A | C | 0.00572403 | 0.66721 | 0.00101927 | 1.70E-08 |
| rs4585442 | A | G | -0.00634685 | 0.688977 | 0.00103625 | 8.10E-10 |
| rs5757675 | G | T | 0.00645515 | 0.259528 | 0.00109902 | 2.70E-09 |
| rs59779556 | T | G | 0.00549133 | 0.553827 | 0.000965961 | 2.00E-08 |
| rs60882754 | A | T | 0.011304 | 0.938985 | 0.00200145 | 1.80E-08 |
| rs7524118 | T | C | -0.0057622 | 0.291624 | 0.00105376 | 4.90E-08 |
| rs7939345 | T | G | 0.00649836 | 0.207569 | 0.0011818 | 4.00E-08 |

**Supplementary Table 5** The summary information for instrumental variables of Daytime sleepiness

| SNP | effect_allele | other_allele | beta | eaf | se | pval |
| --- | --- | --- | --- | --- | --- | --- |
| rs11942333 | G | A | -0.00605348 | 0.675798 | 0.00110327 | 3.80E-08 |
| rs12140153 | G | T | 0.01658 | 0.904572 | 0.00179639 | 2.80E-20 |
| rs12153518 | A | G | 0.00670287 | 0.47224 | 0.00103166 | 6.80E-11 |
| rs13010456 | A | G | 0.00774142 | 0.594693 | 0.00105125 | 2.10E-13 |
| rs13097760 | A | C | -0.00598755 | 0.638696 | 0.00107776 | 3.20E-08 |
| rs13135092 | A | G | -0.0103303 | 0.917193 | 0.0018774 | 3.10E-08 |
| rs1601440 | C | T | 0.00723315 | 0.278583 | 0.00115111 | 4.10E-10 |
| rs17356118 | A | G | -0.00765559 | 0.76879 | 0.00122087 | 2.60E-10 |
| rs1846644 | T | C | -0.0113546 | 0.590849 | 0.00104777 | 2.50E-27 |
| rs285793 | G | A | 0.00676457 | 0.461246 | 0.00103539 | 7.90E-11 |
| rs3122170 | C | A | 0.00950345 | 0.231111 | 0.00122688 | 5.60E-15 |
| rs7598712 | G | T | 0.00579387 | 0.555254 | 0.00104191 | 2.20E-08 |
| rs7607363 | A | G | -0.0060244 | 0.561234 | 0.00103841 | 8.00E-09 |
| rs7837226 | A | G | -0.00574201 | 0.472852 | 0.00103269 | 2.00E-08 |
| rs8015449 | A | G | 0.00619154 | 0.538612 | 0.00103462 | 1.90E-09 |
| rs825127 | T | G | 0.00591296 | 0.530794 | 0.00103275 | 9.50E-09 |
| rs843372 | C | T | 0.00816368 | 0.230053 | 0.00122597 | 2.20E-11 |
| rs886114 | C | T | 0.00604278 | 0.357313 | 0.00107429 | 1.90E-08 |
| rs960986 | C | T | 0.00716034 | 0.636525 | 0.00106799 | 1.50E-11 |
| rs9712275 | C | T | -0.00588405 | 0.485882 | 0.0010319 | 1.30E-08 |

**Supplementary Table6.** The summary information for instrumental variables of frailty

| SNP | effect_allele | other_allele | beta | eaf | se | pval |
| --- | --- | --- | --- | --- | --- | --- |
| rs10891490 | T | C | 0.0188 | 0.4085 | 0.0129 | 2.00E-08 |
| rs12739243 | T | C | 0.0242 | 0.7794 | 0.0051 | 1.28E-09 |
| rs1363103 | T | C | 0.0191 | 0.62 | 0.0038 | 2.23E-08 |
| rs17612102 | T | C | -0.0187 | 0.4067 | 9.00E-04 | 2.85E-08 |
| rs28400568 | A | G | 0.0368 | 0.1109 | 0.0081 | 1.73E-10 |
| rs3959554 | A | G | -0.0189 | 0.5823 | 0.012 | 1.74E-08 |
| rs4146140 | T | C | -0.0198 | 0.3811 | 0.0064 | 6.83E-09 |
| rs4952693 | T | C | -0.0194 | 0.3734 | 0.0032 | 1.47E-08 |
| rs56299474 | A | C | 0.0241 | 0.1733 | 7.00E-04 | 3.94E-08 |
| rs575147125 | T | G | -0.0452 | 0.8301 | 0.0249 | 7.93E-19 |
| rs583514 | T | C | -0.0199 | 0.4889 | 0.0028 | 1.65E-09 |
| rs8089807 | T | C | -0.0248 | 0.1866 | 0.004 | 6.50E-09 |
| rs82334 | A | C | 0.0223 | 0.6823 | 0.0032 | 3.13E-10 |

| **Exposure/Outcome** | **MR-IVW** | | | **MR-Egger** | | | **MR-Egger intercept** | | | **MR-PRESSO** | |
| --- | --- | --- | --- | --- | --- | --- | --- | --- | --- | --- | --- |
|  | **Q** | **Q_df** | **Q_pval** | **Q** | **Q_df** | **Q_pval** | **Intercept** | **SE** | ***P*** val | **Global test Pval** | **Distortion test Pval** |
| Insomnia/frailty | 77.04 | 34 | 3.49e-5 | 76.54 | 33 | 2.59e-5 | 0.13e-2 | 0.27e-2 | 0.64 | 0.17 | NA |
| frailty/ Insomnia | 22.98 | 10 | 0.01 | 15.18 | 9 | 0.09 | 0.49e-2 | 0.23e-2 | 0.06 | 0.07 | NA |
| sleep duration/frailty | 67.26 | 32 | 0.26e-3 | 57.67 | 31 | 0.36e-2 | -0.68e-2 | 0.26e-2 | 0.01 | 0.76e-2 | NA |
| frailty/ sleep duration | 11.03 | 11 | 0.44 | 9.25 | 10 | 0.51 | -0.37E-2 | 0.27e-2 | 0.21 | 0.46 | NA |
| Long sleep duration/frailty | 6.29 | 9 | 0.71 | 4.52 | 8 | 0.80 | -0.71e-2 | 0.53e-2 | 0.22 | 0.72 | NA |
| frailty/ long sleep duration | 18.97 | 12 | 0.09 | 18.34 | 11 | 0.07 | 6.95e-3 | 0.11e-2 | 0.55 | 0.15 | NA |
| short sleep duration/frailty | 234.91 | 22 | 0.30e-2 | 41.82 | 22 | 0.20e-2 | -0.71e-2 | 0.30e-2 | 0.03 | 0.38 | NA |
| frailty/ short sleep duration | 18.56 | 12 | 0.10 | 16.96 | 11 | 0.11 | 0.15e-2 | 0.14e-2 | 0.33 | 0.17 | NA |
| Daytime sleepiness /frailty | 24.15 | 19 | 0.19 | 24.05 | 18 | 0.15 | -0.51e-3 | 0.19e-2 | 0.79 | 0.26 | NA |
| frailty/Daytime sleepiness | 21.05 | 12 | 0.05 | 19.86 | 11 | 0.05 | 0.14e-2 | 0.17e-2 | 0.44 | 0.11 | NA |

**Supplementary Table7.** Heterogeneity test and pleiotropy test after removing SNPs with potential pleiotropy.

**Supplementary Figure S1 sensitivity analysis (A), funnel plot (B), scatter plot (C) and Forest plot (D) of the causal effect of Insomnia on frailty**

**A
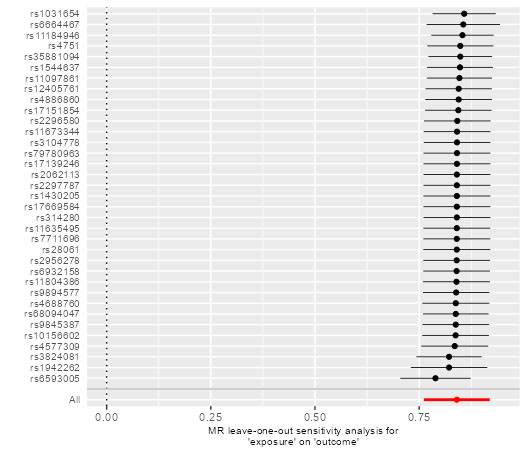
B**
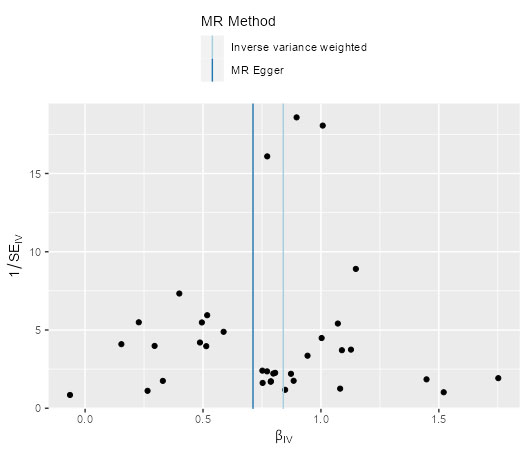


**C
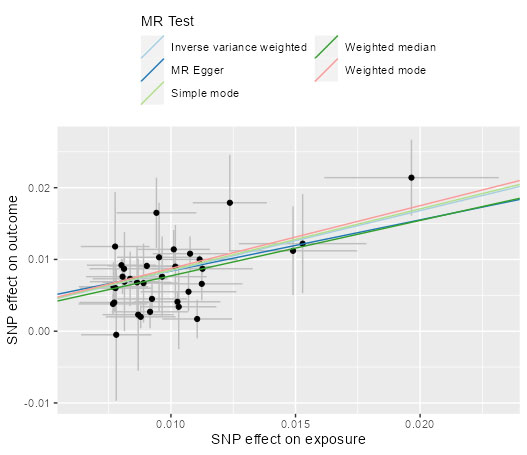

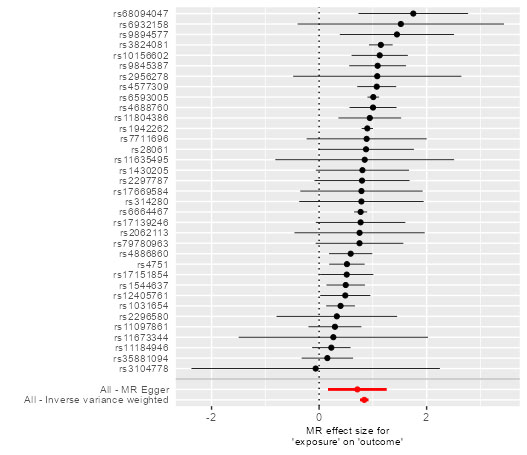
D**

**Supplementary Figure S2 sensitivity analysis (A), funnel plot (B), scatter plot (C) and Forest plot (D) of the causal effect of frailty on Insomnia**

**A
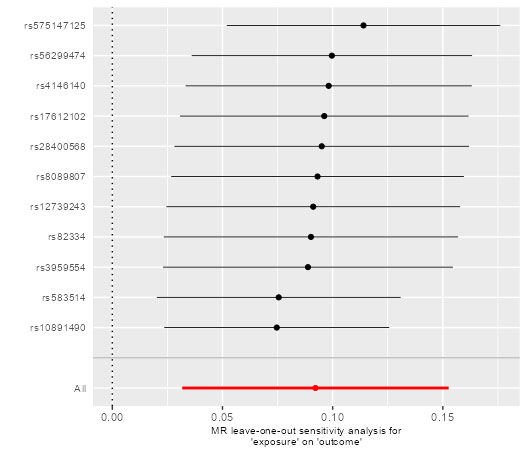

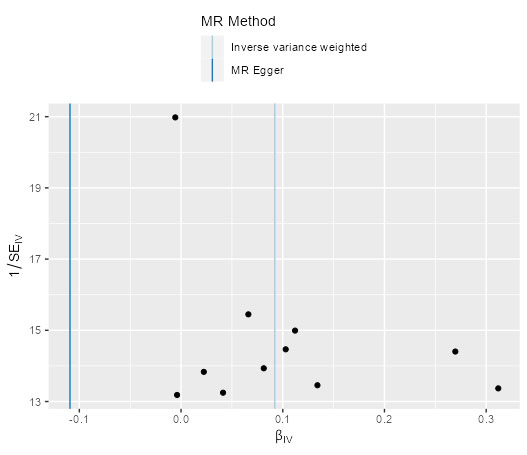
B**

**C
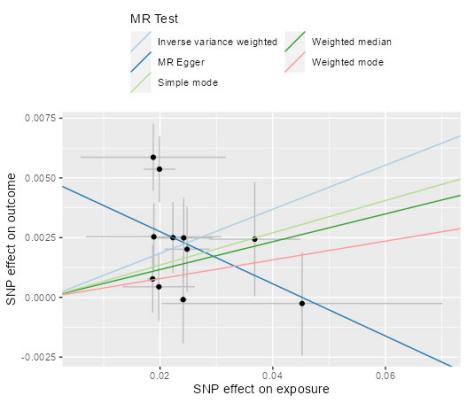

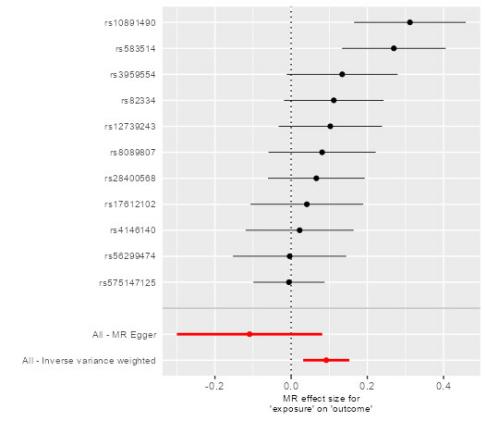
D**

**Supplementary Figure S3 sensitivity analysis (A), funnel plot (B), scatter plot (C) and Forest plot (D) of the causal effect of** **sleep duration on frailty**

**A



B**

**C



D**

**Supplementary Figure S4 sensitivity analysis (A), funnel plot (B), scatter plot (C) and Forest plot (D) of the causal effect of frailty on sleep duration**

**A
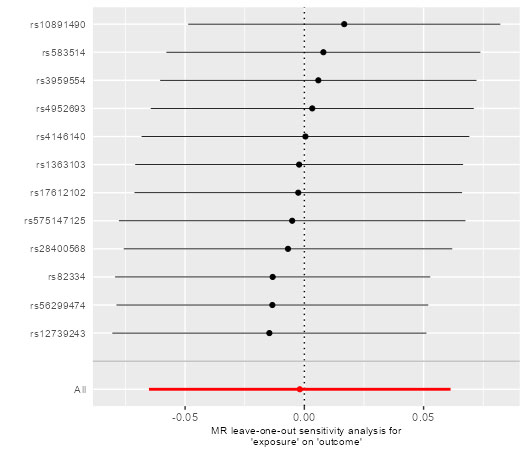

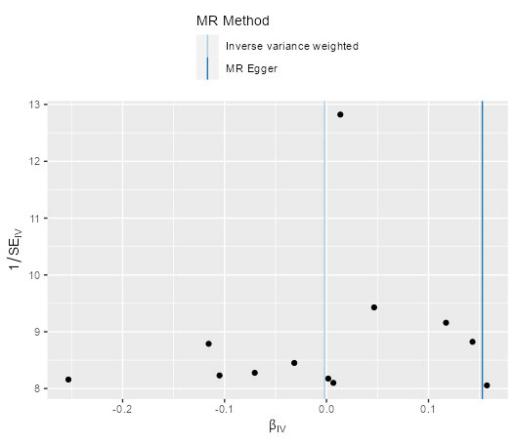
B**

**C
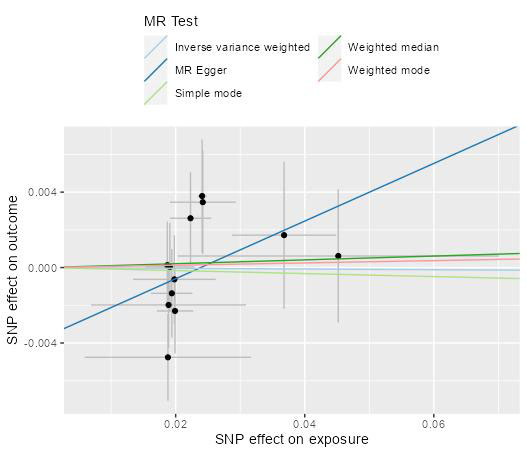

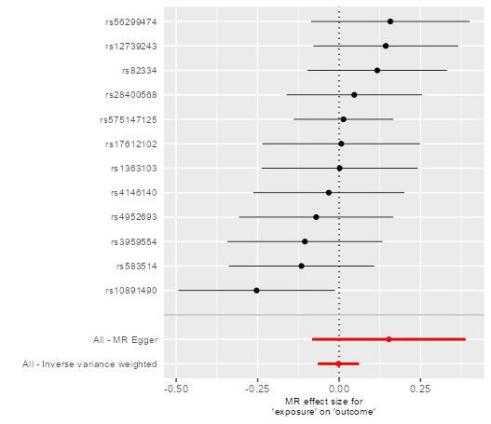
D**

**Supplementary Figure S5 sensitivity analysis (A), funnel plot (B), scatter plot (C) and Forest plot (D) of the causal effect of long sleep duration on frailty**

**A



B**

**C



D**

**Supplementary Figure S6 sensitivity analysis (A), funnel plot (B), scatter plot (C) and Forest plot (D) of the causal effect of frailty on long sleep duration**

**A
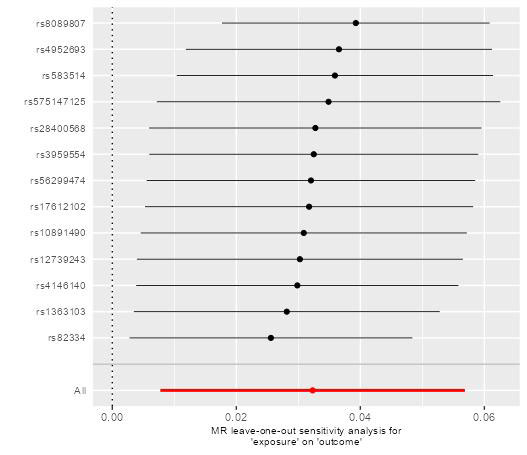

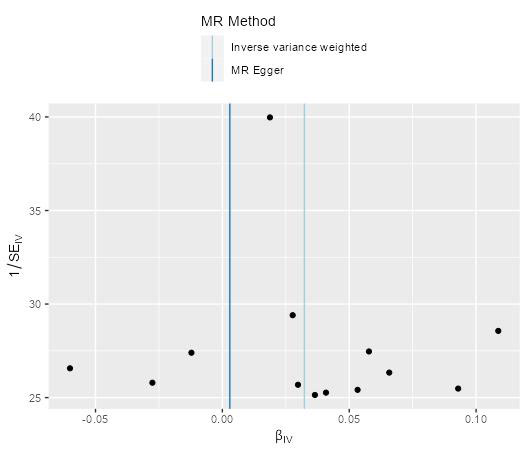
B**

**C
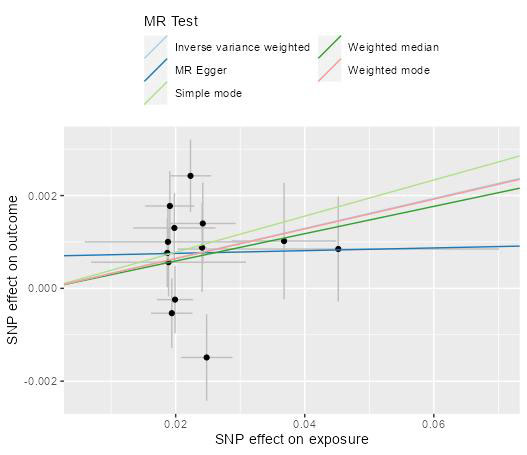

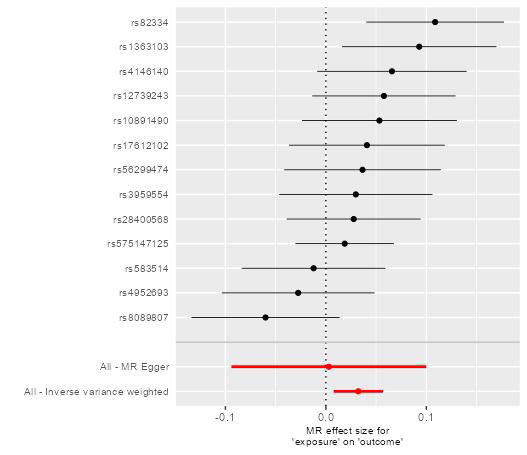
D**

**Supplementary Figure S7 sensitivity analysis (A), funnel plot (B), scatter plot (C) and Forest plot (D) of the causal effect of short sleep duration on frailty**

**A
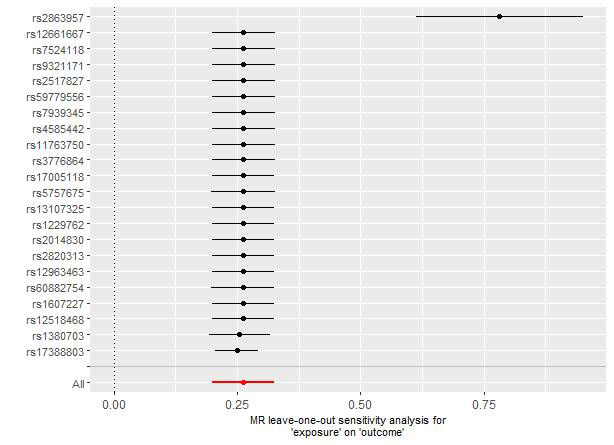

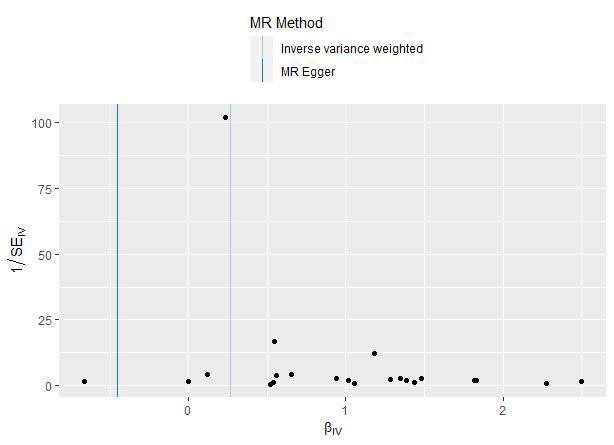
B**

**C
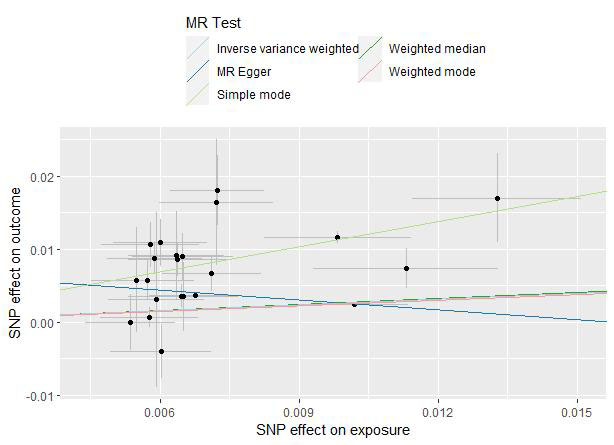

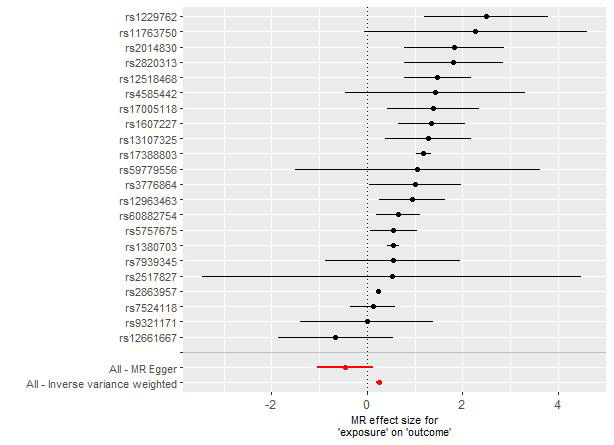
D**

**Supplementary Figure S8 sensitivity analysis (A), funnel plot (B), scatter plot (C) and Forest plot (D) of the causal effect of frailty on short sleep duration**

**A
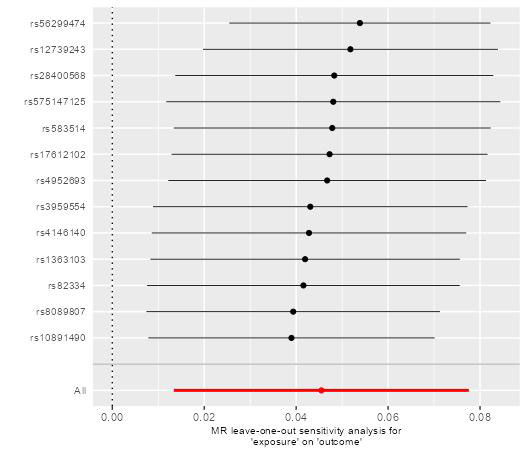

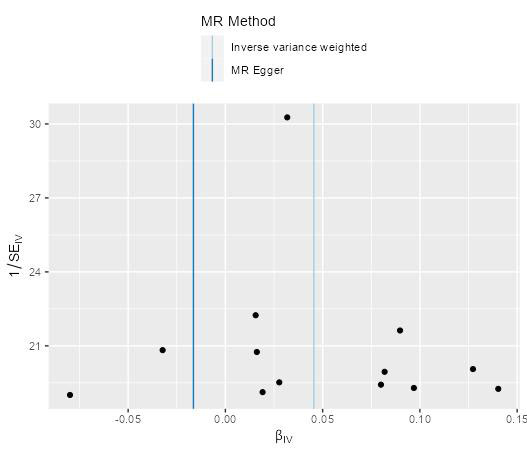
B**

**C
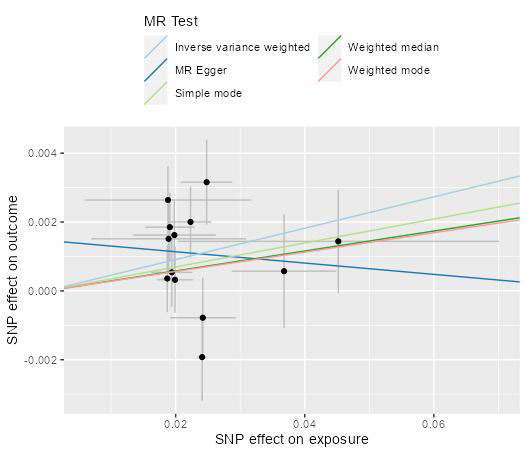

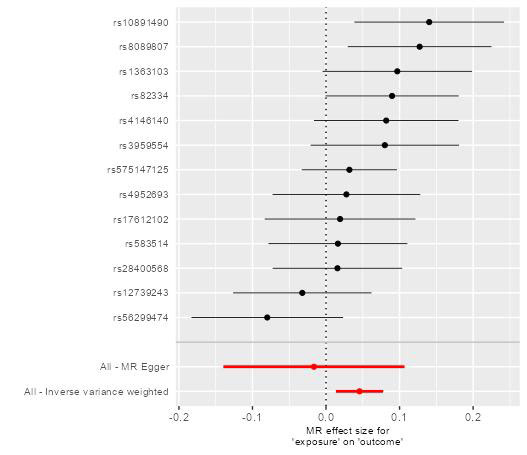
D**

**Supplementary Figure S9 sensitivity analysis (A), funnel plot (B), scatter plot (C) and Forest plot (D) of the causal effect of Daytime sleepiness on frailty**

**A
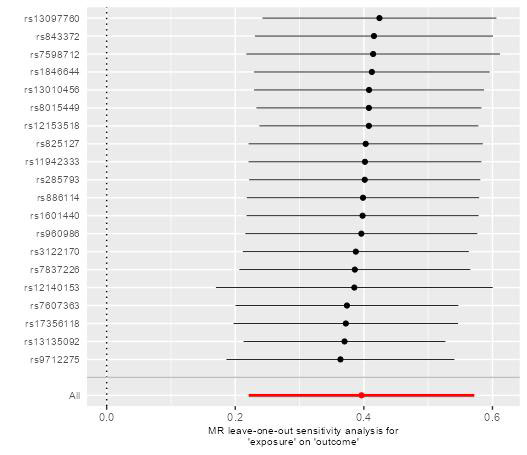

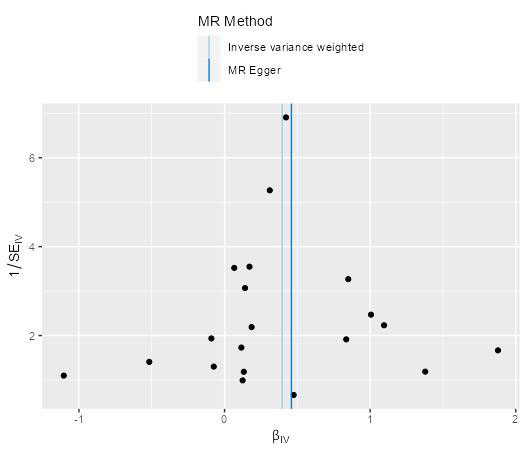
B**

**C
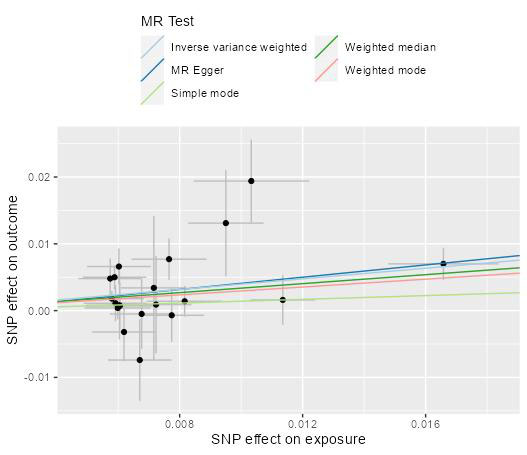

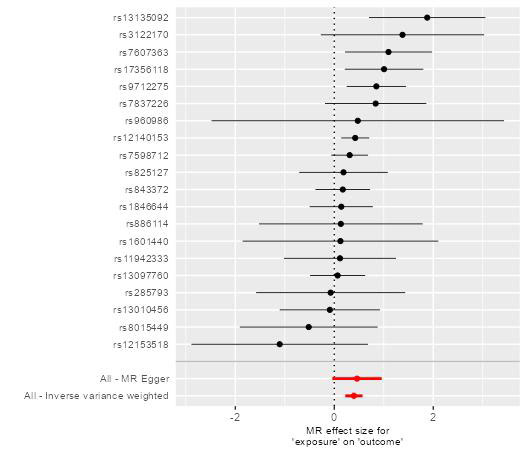
D**

**Supplementary Figure S10 sensitivity analysis (A), funnel plot (B), scatter plot (C) and Forest plot (D) of the causal effect of frailty on Daytime sleepiness**

**A
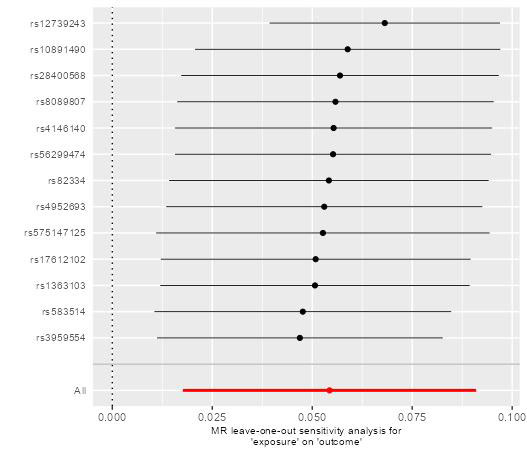

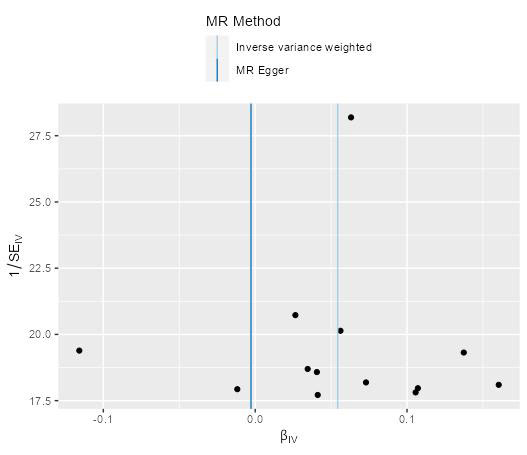
B**

**C
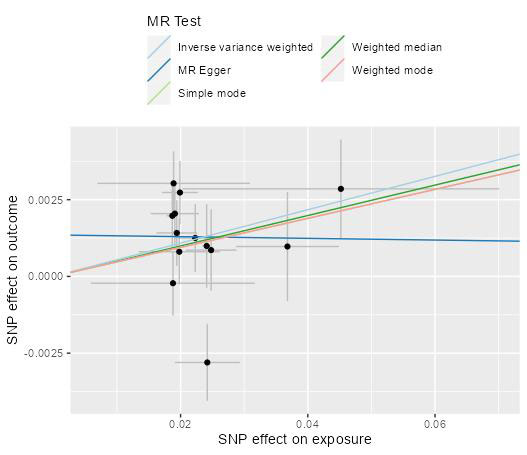

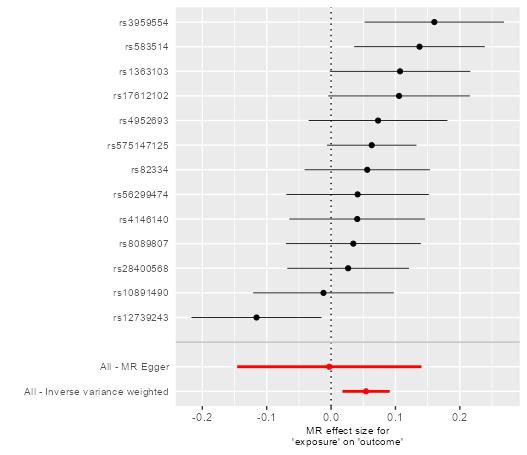
D**
